# New Mitotic Regulators Released from Chromatin

**DOI:** 10.3389/fonc.2013.00308

**Published:** 2013-12-16

**Authors:** Hideki Yokoyama, Oliver J. Gruss

**Affiliations:** ^1^Zentrum für Molekulare Biologie der Universität Heidelberg, DKFZ-ZMBH Alliance, Heidelberg, Germany

**Keywords:** RanGTP, chromatin remodeler, spindle, nuclear pore complex proteins, mitosis, microtubules, microtubule-associated proteins

## Abstract

Faithful action of the mitotic spindle segregates duplicated chromosomes into daughter cells. Perturbations of this process result in chromosome mis-segregation, leading to chromosomal instability and cancer development. Chromosomes are not simply passengers segregated by spindle microtubules but rather play a major active role in spindle assembly. The GTP bound form of the Ran GTPase (RanGTP), produced around chromosomes, locally activates spindle assembly factors. Recent studies have uncovered that chromosomes organize mitosis beyond spindle formation. They distinctly regulate other mitotic events, such as spindle maintenance in anaphase, which is essential for chromosome segregation. Furthermore, the direct function of chromosomes is not only to produce RanGTP but, in addition, to release key mitotic regulators from chromatin. Chromatin-remodeling factors and nuclear pore complex proteins, which have established functions on chromatin in interphase, dissociate from mitotic chromatin and function in spindle assembly or maintenance. Thus, chromosomes actively organize their own segregation using chromatin-releasing mitotic regulators as well as RanGTP.

## Introduction

In all organisms, chromosomes have to be segregated faithfully to daughter cells to stably transmit genetic information. Perturbation of this process can result in chromosome mis-segregation, which has a clear link to aneuploidy and cancer development in humans. For a long time, chromosomes were thought to be passengers segregated by spindle microtubules. But it is now evident that chromosomes play a major role in their own segregation during mitosis. The active role of chromatin was initially shown by an experiment, in which injection of bacteriophage lambda DNA into *Xenopus* eggs, arrested in metaphase, turned out to be sufficient to trigger MT formation (1). The “chromatin effect” was reproduced with plasmid DNA-coated beads incubated in *Xenopus* egg extracts. DNA-beads were converted into functional chromatin in extracts and induced spindle assembly (2). The chromatin-beads contained neither centrosomes nor kinetochores, showing that chromatin is sufficient to drive spindle assembly.

**Figure 1:**
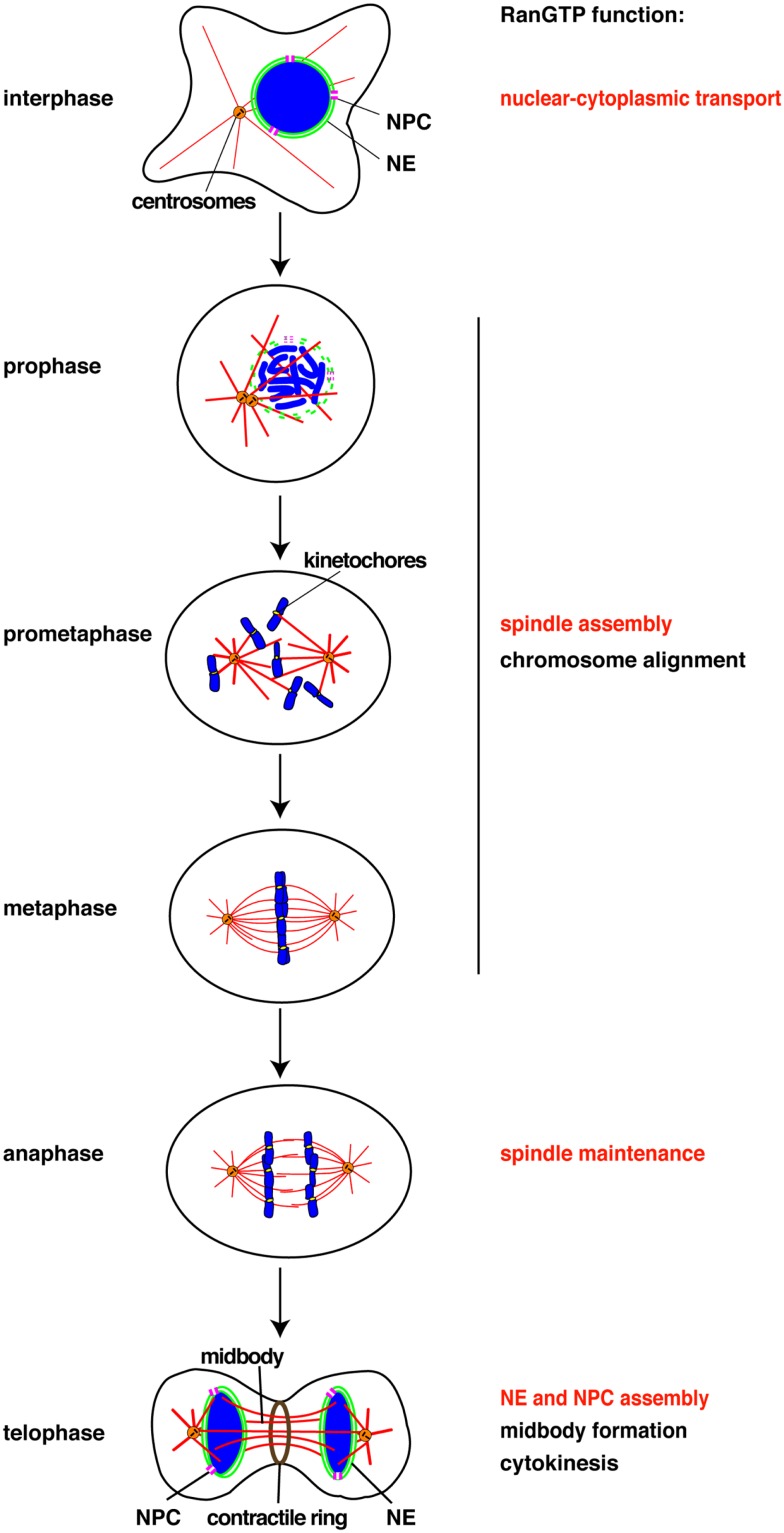
**Chomatin and RanGTP function at each cell cycle stage**. RanGTP directly stimulates the mitotic events displayed in red. Other events (black) may also be RanGTP-regulated, but have not yet been studied. NE, nuclear envelope, NPC, nuclear pore complex.

The Ran GTPase was identified as a factor essential for chromatin-driven spindle assembly (3–7). The guanine nucleotide exchange factor for Ran (RCC1) localizes to chromatin while the GTPase activating protein for Ran (RanGAP) resides in the mitotic cytoplasm. The specific localizations of RCC1 and RanGAP result in high concentrations of the GTP bound form of Ran (RanGTP) locally around chromosomes. RanGTP binds to the heterodimeric nuclear transport receptor importin α/β and dissociates nuclear localization signal (NLS)-containing proteins from the importins (8–10). Liberated NLS proteins function in spindle assembly around chromosomes (Figure 1).

Several NLS proteins have since been identified as RanGTP-dependent spindle assembly factors (11). Among them, proteins such as NuMA do not have any reported functions in interphase, indicating that their nuclear localization separates them from microtubules in the cytoplasm. When the nuclear envelope breaks down upon mitotic entry, NuMA stimulates microtubule nucleation in a RanGTP-dependent manner (9, 10). It has been recently discovered, however, that some of the NLS proteins with established interphase functions play distinct roles in mitosis, including proteins dissociating from mitotic chromatin. Here we summarize this new class of NLS proteins as “chromatin-releasing mitotic regulators.”

## Chromatin-Binding and Dissociation upon Mitotic Entry

Chromatin structure dramatically changes at the onset of mitosis, with the formation of highly ordered and condensed chromosomes. Some chromatin-binding proteins like condensin complex proteins specifically bind to mitotic chromatin while others, like cohesin components, dissociate (12). RCC1 binds to chromatin throughout the cell cycle, but more strongly in mitosis due to several mechanisms including its mitosis-specific phosphorylation by Cdk1 (13). Increased binding of RCC1 to chromatin is essential for producing a high RanGTP concentration around chromosomes and for spindle assembly. In parallel, it has become clear that some of the chromatin-binding proteins that dissociate during mitosis play important, mitosis-specific roles (Table 1).

**Table 1:** **Chromatin-releasing mitotic regulators**.

Protein	Interphase localization	Interphase function	Mitosis localization	Mitotic function	*In vitro*	RanGTP regulation
ISWI	Nucleus, chromatin (12, 14)	Unknown, no effect on chromatin decondensation and DNA replication (12, 14)	The spindle, enrichment on poles and chromosomes (15)	Spindle maintenance, RanGTP-dependent microtubule stabilization in anaphase (15); no effect on chromosome condensation and sister chromatid cohesion (12)	Chromatin-remodeling (44); RanGTP-dependent MAP, inhibited by importin α/β (15)	Yes (15)
Ino80	Nucleus (45); chromatin (20)	Transcription (17–19); DNA repair (46); DNA replication (47)	Spindle microtubules (20)	Spindle assembly (20)	Chromatin-remodeling (48); MAP (21)	Not determined
Pontin/RuvBL1	Nucleus, chromatin (49)	Transcription, DNA repair (50)	Spindle poles and microtubules (22, 49)	Spindle assembly, interact with γ-tubulin (22)	Chromatin-remodeling (48)	Not determined
Rae1/Gle2	NPCs (27)	mRNA export through the NPC (27)	The spindle, enrichment on poles chromosomes (26)	Spindle assembly, functions as a rebonucleoprotein complex, RanGTP-dependent microtubule nucleation/stabilization (26)	MAP (26)	Yes (26)
Nup98	NPCs (51)	NPC assembly, essential for assembling the permeability barrier (52)	The mitotic cytoplasm (53)	Spindle assembly, counteract MCAK (28)	Recombinant Nup98 forms the permeability barrier (52); MAP (28)	Not determined
Nup107-160 complex	NPCs (54, 55)	NPC assembly (54, 55)	Kinetochores and spindle microtubules (29, 30)	Spindle assembly in *Xenopus* egg extracts (30); γ-tubulin recruitment and microtubule nucleation at unattached kinetochores in human cells (31)	Immunoprecipitates from *Xenopus* egg extracts nucleate microtubules *in vitro* in RanGTP-dependent manner (31)	Yes (31)
RanBP2/Nup358	NPCs (56, 57)	Nuclear transport through the NPC (58)	Kinetochores and spindle microtubules (59)	Required for microtubule-kinetochore interaction (32, 33); recruited to kinetochores dependent on RanGTP and Crm1 (34)	Protein sumoylation (60)	Yes (34)

## Chromatin-Remodeling Factors

The chromatin-remodeling ATPase ISWI binds chromatin during interphase, although its specific role in the nucleus remains unclear (12, 14). The majority of ISWI dissociates from mitotic chromatin and re-localizes to the spindle (15). ISWI directly binds microtubules in a RanGTP-dependent manner. The region that contains chromatin-binding domains and an NLS also mediates microtubule-binding of ISWI. ISWI is, however, not required for spindle assembly, but is essential for maintaining spindle microtubules during anaphase and in turn for chromosome segregation (15) (Figure 1). This microtubule stabilizing function of ISWI is independent of chromatin-remodeling. The release from chromatin is a prerequisite for ISWI to function as a microtubule-associated protein (MAP), but its microtubule-binding is further regulated by RanGTP.

Chromatin-remodeling ATPases share a SWI/SNF-type ATPase domain, but are classified into four different families (SWI/SNF, ISWI, INO80, and CHD) according to unique flanking domains (16). The INO80-family ATPase (Swr1 and Ino80)-containing complexes incorporate and remove the histone variant H2A.Z into the first nucleosome of genes, respectively, regulating gene expression (17–19). However, Ino80 associates with spindle microtubules during mitosis, and its depletion from human cells leads to defective spindle assembly and abnormal chromosome segregation (20). Ino80 directly binds to microtubules *in vitro* (21). It has not been determined if the Ino80 function in microtubule regulation is regulated by the RanGTP system. Pontin/RuvBL1, a component of the Ino80-containing chromatin-remodeling complex, was shown to localize at spindle poles and microtubules, to interact with γ-tubulin, and to be essential for spindle assembly (22).

The SWI/SNF-family ATPases, BRM, and BRG-1, are phosphorylated during mitosis and released from condensed chromosomes (23). It has been suggested that their dissociation could be the mechanism leading to transcriptional arrest during mitosis. Other research demonstrated that BAF180, a component of BRG-1-containing chromatin-remodeling complex, localizes at spindle poles and kinetochores (24), suggesting that the complex could participate in spindle regulation and cell division.

Taken together, these data indicate that there are general roles for chromatin-remodeling ATPases and complexes (16) to regulate microtubules in mitosis. Chromatin-remodeling alters nucleosome positions, evicts histones, or incorporates histone variants during DNA damage repair and DNA replication, maintaining genomic integrity (25). These efforts would be irrelevant if chromosomes were not properly segregated during mitosis. Chromatin-remodeling factors regulate spindle microtubules independent of chromatin-remodeling, but this regulation is for chromosome segregation and still serves to maintain genomic integrity.

## Nuclear Pore Complex Proteins

The nuclear pore complex (NPC), composed of ~30 different proteins (nucleoporins or Nups), is critical to transport protein and RNA across the nuclear envelope in interphase (Figure 1). The envelope breaks down during mitosis in metazoan cells and the NPC disassembles into nucleoporin subcomplexes and dissociates from chromatin. It has been shown that some nucleoporins play important roles in mitosis (Table 1).

The nucleoporin Rae1/Gle2 directly binds microtubules *in vitro*, localizes to the mitotic spindle, and is required for spindle assembly (26). A Rae1-containing ribonucleoprotein complex nucleates or stabilizes microtubules in a RanGTP/importin β-regulated manner (26). Rae1 is known to form a nucleoporin subcomplex with Nup98 (27). Nup98 was also shown to directly bind microtubules as well as MCAK, a microtubule depolymerizing kinesin motor protein (28). Although localizing mostly in the mitotic cytoplasm, Nup98 is required for spindle assembly by antagonizing MCAK (28). It remains unclear if Rae1 and Nup98 function together or independently in spindle assembly.

The Nup107-160 complex, the largest subcomplex of the NPC, re-localizes to kinetochores and spindle microtubules during mitosis (29, 30). The components of the Nup107-160 complex are maintained in interphase and mitosis (30). Using *Xenopus* egg extract, it was shown that the Nup107-160 complex is required for spindle assembly independent of its interphase function at nuclear pores (30). In extracts depleted of the Nup107-160 complex, microtubules are nucleated from sperm centrosomes normally but eventually disassembled, leaving largely unattached sperm chromosomes (30). The molecular function of the Nup107-160 complex in spindle assembly remains unclear. When microtubule-kinetochore attachment is disrupted (e.g., by nocodazole treatment), the Nup107-160 complex recruits the γ-tubulin ring complex (γTuRC) to kinetochores and promotes microtubule nucleation there (31). Whether the complex recruits γTuRC to kinetochores and microtubules under physiological conditions is an open question.

RanBP2/Nup358 also re-localizes from NPCs to kinetochores and spindle microtubules and plays an essential role in microtubule-kinetochore interaction (32, 33). RanBP2 depletion in human cells by RNAi causes loss of stable kinetochore microtubules and accumulation of mitotic cells with multipolar spindles and unaligned chromosomes (33). The kinetochore localization of RanBP2 is dependent on RanGTP and the nuclear export factor Crm1 (34). However, the molecular function of RanBP2 at kinetochores remains to be determined.

## Spatiotemporal Control of the Mitotic Regulators Released from Chromatin

The above findings demonstrate that chromatin-remodeling factors and nucleoporins play important, distinct roles during mitosis (Table 1). The mechanism by which these proteins dissociate from mitotic chromatin, however, remains largely unknown. Chromatin structures dramatically change during mitosis, including chromatin condensation, and these modifications may cause the release of certain chromatin-binding proteins (12). Another possibility is that chromatin proteins themselves may be regulated during mitosis by posttranslational modifications or/and interacting proteins. The phosphorylation of BRM and BRG-1, for instance, correlates with their dissociation from mitotic chromatin (23). Phosphorylation of Nup98 is crucial for NPC disassembly (35) and may lead to the dissociation of multiple nucleoporins from chromatin. Thus, chromatin either actively releases chromatin-binding proteins or makes use of proteins leaving chromatin for mitotic organization.

Dissociation from chromatin seems to be sufficient to activate chromatin proteins. In the mitotic cytoplasm, however, importins bind to NLS proteins and inhibit some of the proteins, while not affecting others (36). RanGTP locally activates the inhibited proteins around chromosomes. Thus, mitotic regulators that dissociate from chromatin function either locally around chromosomes or throughout the mitotic cytoplasm. Accumulating evidence indicates that this spatial regulation of individual proteins is critical for proper mitotic progression.

While metazoan cells segregate chromosomes in the mitotic cytoplasm after disassembling nuclear envelope and NPC (open mitosis), protozoa such as yeasts segregate inside the nucleus without disassembling envelope and NPC (closed mitosis) (37). In yeasts, RanGTP is indirectly required for spindle assembly by mediating nuclear import of the microtubule regulator Alp7/TACC (38). Therefore, the mitotic regulators that dissociate from chromatin and NPCs seem to have evolved in metazoa with the disassembly of the nuclear envelope and NPCs.

## New Mitotic Functions of Chromosomes Beyond Spindle Assembly

Chromosomes drive spindle assembly in early mitosis and later, through the function of ISWI, maintain spindle microtubules in anaphase (15) (Figure 1). ISWI depletion from *Xenopus* egg extracts or *Drosophila* cultured cells does not affect spindle assembly, but upon anaphase entry spindle microtubules depolymerize and chromosomes do not segregate (15). This finding suggests that chromosomes may regulate multiple mitotic events essential for their own segregation. Indeed, perturbation of the Ran pathway in *Drosophila* embryos by injecting recombinant proteins induced defects in mitotic spindle assembly, in chromosome alignment to the metaphase plate, in chromosome segregation, and in the assembly of the microtubule midbody (39). RNAi of components of the Ran pathway in *C. elegans* also resulted in multiple mitotic defects (40). Although these experiments did not discriminate between direct and indirect effects of the Ran pathway, they suggest possible new roles of chromosomes in their own alignment and in midbody formation (Figure 1).

Currently, all the known RanGTP targets are MAPs or their regulators (11). It has been shown in starfish oocytes that chromatin drives local actin polymerization to mediate chromosome congression (41). The MAP NabKin has been identified as a nuclear protein that binds fibrous actin in a RanGTP-dependent manner (42). NabKin localizes to the mitotic spindle as well as to cortical fibrous actin in *Xenopus* oocytes, and is essential for cytokinesis (42), although the molecular function of NabKin remains to be determined. Chromosomes and RanGTP thus also seem able to regulate the actin cytoskeleton in chromosome congression/alignment and cytokinesis (Figure 1).

Finally, RanGTP drives the assembly of a lamin B “matrix” around chromosomes in mitosis (43). The matrix is essential for proper spindle assembly in human cells and *Xenopus* egg extracts, in particular for the organization of assembled microtubules into bipolar spindles (43). The mechanism of lamin B matrix assembly is not understood but is certainly independent of microtubule assembly. It will be important to identify further RanGTP targets involved in the assembly of microtubules, actin filaments, and the lamin matrix.

## Conclusion and Perspective

Chromosomes induce the production of RanGTP in their vicinity, which is essential for mitotic progression. Here we propose the release of chromatin-bound mitotic regulators as the second key role of chromosomes. Released proteins function independently of chromatin during mitosis with or without spatial control by RanGTP. The list of the chromatin-releasing mitotic regulators seems certainly to increase in the future. Clarification of the role of each protein will significantly contribute to our understanding of how chromosomes regulate their own segregation.

## Conflict of Interest Statement

The authors declare that the research was conducted in the absence of any commercial or financial relationships that could be construed as a potential conflict of interest.
